# Gastric mucosa-associated lymphoid tissue lymphoma in conjunction with multiple lymphomatous polyposis in the context of *Helicobacter pylori* and *Helicobacter suis* superinfection

**DOI:** 10.1007/s12328-020-01310-5

**Published:** 2021-01-04

**Authors:** Toshikatsu Naito, Ryo Yuge, Shinji Tanaka, Rina Otani, Hiroki Kadota, Hidehiko Takigawa, Tadamasa Tamura, Kazuhiro Sentani, Wataru Yasui, Yasuhiko Kitadai, Kazuaki Chayama

**Affiliations:** 1 Department of Gastroenterology and Metabolism, Hiroshima University, Hiroshima, Japan; 2 Department of Endoscopy, Hiroshima University, 1-2-3, Kasumi, Minami-ku, Hiroshima, Japan; 3 Department of Gastroenterology, Tsuchiya General Hospital, Hiroshima, Japan; 4 Department of Molecular Pathology, Hiroshima University, Hiroshima, Japan; 5 Department of Health Science, Prefectural University of Hiroshima, Hiroshima, Japan

**Keywords:** Mucosa-associated lymphoid tissue lymphoma, Non-*H. pylori helicobacters*, Multiple lymphomatous polyposis

## Abstract

A 53-year-old woman visited a doctor and complained of chest discomfort after meals. Esophagogastroduodenoscopy showed multiple granular elevations in the gastric body. After biopsies from the elevations, she was diagnosed with mucosa-associated lymphoid tissue (MALT) lymphoma. Polymerase chain reaction also detected *Helicobacter pylori* and *H. suis*. Treatment to eradicate *H. pylori and H. suis* was successful. Endoscopic examination after the bacterial eradication treatment showed that multiple granular elevations remained in the gastric body; however, no lymphoma cells were found during histopathological examination. Thus, we reported a case of *H. pylori*-positive gastric MALT lymphoma with a unique morphology associated with *H. suis* superinfection*.*

## Introduction

Mucosa-associated lymphoid tissue (MALT) lymphoma is a type of low-grade B-cell non-Hodgkin lymphoma. Classic signs of gastric MALT lymphoma include depressed lesions with faded coloring and indistinct boundaries. Elevated lesions are rare, and, to our knowledge, there are no reports of the multiple lymphomatous polyposis type (MLP-type) with multiple granular elevations in the gastric body.

*Helicobacter pylori* is associated with the occurrence of gastric MALT lymphoma [1]; nevertheless, recent reports have associated non-*H. pylori helicobacters* (NHPH) with gastric MALT lymphoma. In particular, *H. suis* is a frequent cause of infection in cases of gastric MALT lymphoma with a nodular gastritis-like appearance [2]. Here we report a case of *H. pylori*-positive gastric MALT lymphoma with a unique morphology associated with *H. suis* superinfection.

## Case report

**Fig. 1:**
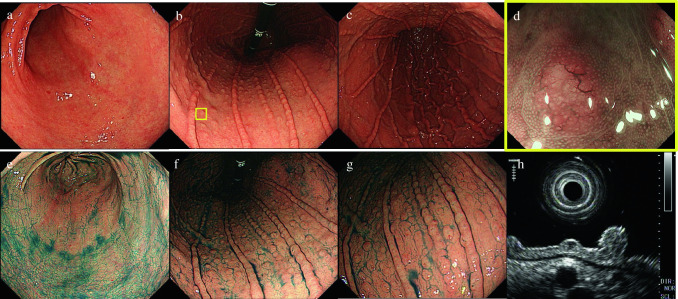
EGD at first visit (before bacterial eradication). **a** Inconspicuous granular elevations can be seen in the vestibular area, **b**, **c** with multiple granular elevations in the body. **d** Narrow-band-imaging shows dilated blood vessels at the surface layer of the elevations. **e** Spraying of indigo carmine shows no elevations in the vestibular area. **f**, **g** Highly distinct granular elevations can be seen in the body. **h** In endoscopic ultrasonography, the lesions are extracted as hypoechoic masses on the surface of the second layer. *EGD* esophagogastroduodenoscopy

**Fig. 2:**
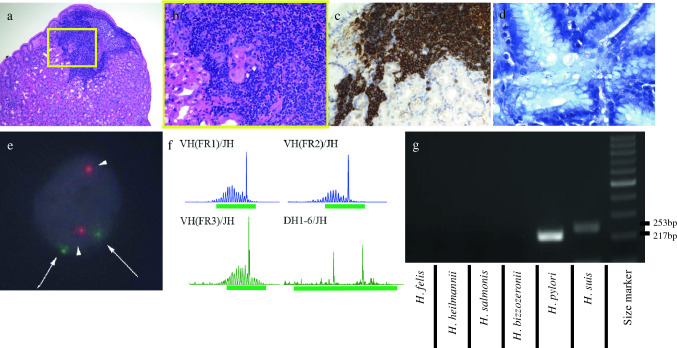
Pathological findings and other results (before bacterial eradication). **a** There is atypical cell (mainly small-to-medium lymphocytes including mild nuclear constriction in the lamina propria) invasion (HE staining at × 100 magnification). **b** Some lymphoepithelial lesions can be seen (HE staining at 400 × magnification). **c** Immunohistochemical findings (before bacterial eradication). Tumor cells are positive for CD20. **d** Gimenez staining shows helical bacilli in the deep gastric glands. **e** FISH does not reveal the API2-MALT1 gene in the lesions. **f** IgH gene rearrangement detects a single peak in four out of five areas. Red, MALT1 signal; green, API2 signal **g** PCR assay (before bacterial eradication). *H. pylori* (217 bp) and *H. suis* (253 bp) are detected, with negativity for other NHPH. *HE* hematoxylin and eosin, *FISH* fluorescence in situ hybridization, *PCR* polymerase chain reaction, *NHPH* non-*H. pylori helicobacters*

**Fig. 3:**
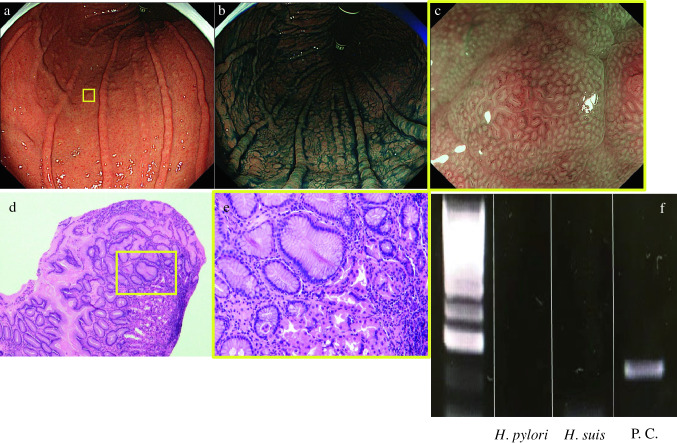
EGD, pathological findings and PCR assay (after bacterial eradication). **a**, **b** EGD (after bacterial eradication) shows multiple granular elevations remaining in the gastric body. **c** Disappeared dilated blood vessels on the surface layer of the elevations. **d**, **e** Pathological analysis (after bacterial eradication) shows no remaining lymphoma cells (**d** HE staining at × 100 magnification, **e** HE staining at × 400 magnification). **f** PCR assay (after bacterial eradication) shows negativity for *H. pylori* and *H. suis*. *PC* positive control, *HE* hematoxylin and eosin, *EGD* esophagogastroduodenoscopy, *PCR* polymerase chain reaction

A 53-year-old woman visited a doctor and mainly complained of chest discomfort after meals. Esophagogastroduodenoscopy (EGD) showed multiple granular elevations in the gastric body. She was referred to our hospital for further examination. Her abdomen was flat and soft with no abnormal physical findings. Her medical history included angina and endometriosis, and she owned a pet cat, which is known to be a natural host for NHPH. Blood tests revealed no significant results. EGD showed closed-type atrophic changes in the background mucosa, with multiple granular elevations measuring 2–3 mm centered in the gastric body (Fig. 1a-c, e–g). A small depression was present at the tip of the elevations. Magnified narrow-band imaging showed dendritic dilated blood vessels on the surface layers of all elevations (Fig. 1d). In ultrasonic endoscopy, the lesions were extracted as hypoechoic masses in the shallow part of the second layer (Fig. 1h). Biopsies obtained from multiple elevations showed invasion of atypical cells and some scattered lymphoepithelial lesions and small-to-medium lymphocytes with mild nuclear constriction, even in the lamina propria (Fig. 2a, b). No lymphoma cell infiltration was observed in biopsy tissue from the background mucosa. Immunohistochemical analysis showed that the lesions were positive for CD20 (Fig. 2c), CD79a, and bcl-2 and negative for CD10 and cyclinD1. B-cell markers predominated the stain images, and the patient was diagnosed with MALT lymphoma. Fluorescence in situ hybridization (FISH) showed negative results for the API2-MALT1 gene (Fig. 2e), and IgH gene rearrangement detected a single peak in four out of five areas (Fig. 2f). The urea breath test did not show a high level (approximately 2.2‰) although serum *H. pylori* IgG antibodies were positive and *H. pylori* antigen was found in her stool. Because she lived with a cat and her endoscopic findings were atypical for gastric MALT lymphoma, we suspected NHPH infection. Polymerase chain reaction (PCR) was performed using biopsy specimens for *H. pylori* and five strains of NHPH (*H. suis, H. bizzozeroni, H. salmonis, H. felis, H. heilmannii*) that typically infect humans (Table 1) [3], and *H. pylori* and *H. suis* were detected (Fig. 2g). Gimenez staining of the biopsy tissue did show helical bacilli; however, differences from *H. pylori* were histologically indistinct, and it was difficult to make a definitive histopathological diagnosis of the NHPH (Fig. 2d). No lesions outside the stomach were found in colonoscopy, small bowel capsule endoscopy, and positron emission tomography–computed tomography. The patient was diagnosed with stage I lymphoma as per the international Lugano classification [4]. Primary treatment involved 7-day bacterial eradication therapy using three oral agents: vonoprazan fumarate, amoxicillin hydrate, and clarithromycin. Subsequently, her stool tested negative for *H. pylori* antigen, and the bacterial eradication therapy was considered successful. Endoscopic images at 6 months after bacterial eradication showed that multiple granular elevations remained in the gastric body; however, the dilated blood vessels found in the surface layer had disappeared (Fig. 3a–c). Histopathologically, no lymphoma cells remained (Fig. 3d, e). PCR with gastric mucosal tissue after the bacterial eradication treatment confirmed that *H. pylori* and *H. suis* had been successfully eradicated (Fig. 3f). Follow-up observation with regular endoscopic examination was planned to check for the recurrence of gastric MALT lymphoma or any changes in the form of the granular elevations.

**Table 1:** Primers for detection of *H. pylori* and non-*H. pylori Helicobacters*

NHPH	Primer (F: forward, R: reverse)	Size (bp)
*H. pylori*	F: AAAGAGCGTGGTTTTCATGGCG	217
	R: GGGTTTTACCGCCACCGAATTTAA	
*H. suis*	F: CACCACCCCGGGGAAGTGATCTTG	253
	R: CTACATCAATCAAATGCACGGTTTTTTCTTCG	
*H. bizzozeronii*	F:CGCTTTGAACCCGGTGAGAAAA	172
	R:TATCGCAACCGCAATTCACAACA	
*H. felis*	F:TCCCACTACCGGGGATCGTG	350
	R:CAGCGGTTACAATCAAGCCCTCA	
*H. salomonis*	F:CTTTGGGTCTGTGCCTGCCTG	219
	R:CATCGCGGATAGTCTTACCGCCT	
*H. heilmannii* s.s	F:CTTTCTCCTGGTGAAGTGATTCTC	368
	R:CAGTTGATGGTGCCAAAG

## Discussion

Members of the genus *Helicobacter* other than *H. pylori* are referred to as NHPH. These can infect the gastric mucosa in humans, resulting in gastritis. They have also been implicated in the occurrence of gastric MALT lymphoma. In addition, reports on the pathogenicity of NHPH include acute gastritis. Asymptomatic chronic gastritis of the vestibular area is common [5]. Morgner et al*.* reported that NHPH associated with gastric diseases in humans include at least five strains, namely *H. suis, H. bizzozeroni, H. salmonis, H. felis*, and *H. heilmannii,* and that NHPH has a stronger association with the occurrence of gastric MALT lymphoma [6]. *H. suis* infection is also frequently present in gastric MALT lymphomas associated with NHPH infections [7]. However, multiple forms of small elevated lesions have been reported in cases of *H. suis*-infected gastritis, including nodular gastritis and nodular gastritis-like MALT lymphoma. This suggests that small elevated lesions tend to be more frequent in *H. suis*-infected gastritis. It has been reported that *H. suis* infection usually causes hyperplasia of lymphatic follicles [8, 9], which could be one reason for the small elevated lesions. Therefore, we believe that *H. suis* infection may be responsible for the multiple granular elevations observed in the present case. Unlike the “cobblestone-like” or “nodular” morphology previously observed in the vestibule in cases of nodular gastritis-like MALT lymphoma [2, 10], multiple smaller granular elevations were observed in the gastric body in the present case. After consultation with a gastroenterologist experienced in dealing with cases of MALT lymphoma and a pathologist with extensive experience in the pathological diagnosis of MALT lymphoma, the present case was classified as the MLP type. There are some reports of MLP-type MALT lymphoma in the small and large intestines [11, 12]; however, as per our search of the PubMed database, there are no published reports of MLP-type MALT lymphoma in the stomach.

Superinfection with NHPH has been reported in 6.5% patients with *H. pylori* infection [13]. Although a certain number of cases of *H. suis* and *H. pylori* superinfection are expected, there is no report involving MLP-type lesions. Thus, factors other than superinfection, such as other microbial infections, environmental factors, and genetic factors, may have contributed to the peculiar distribution and morphology of the lesions in this case. However, the involved factors have not been elucidated, and accumulation of similar cases is necessary to the clarify them.

In the present case, FISH showed negative results for the API2-MALT1 gene. No association between the API2-MALT1 chimera gene and bacterial infection has been reported.

Natural hosts of NHPH include dogs, cats, and pigs; therefore, there is a possibility of zoonotic infection in our case. Consequently, it is important to inquire about any history of pets in such cases [14]. The patient in the present report had a cat at the time of her diagnosis, which could be a possible source of the NHPH infection. She had also raised several other pet animals in the past, and it was not possible to determine when the infection occurred and when MALT lymphoma developed.

Diagnosis of NHPH infection may involve methods used to diagnose *H. pylori* infections, such as pathological observation, the urea breath test, the rapid urease test, culture, fecal antigen tests, or urinary antibody tests. However, these methods have not been fully established. Although pathological observation is an effective method, it is mostly impossible to confirm the bacteria in the biopsy tissue because NHPHs are quite unevenly distributed. Even if spiral bacteria are observed, *H. pylori* may also be present in the form of spiral bacteria, and it cannot be differentiated from NHPH [15]. Currently, PCR is the standard form of identification [3]. In the present case, PCR enabled the confirmation of *H. pylori* and *H. suis* infections. PCR is also useful for confirming the status of *H. suis* after bacterial eradication treatment [16]; therefore, it is an important test for follow-ups over the course of bacterial treatment. A limited number of facilities are capable of using PCR; simpler and easier methods of diagnosing infections should be established.

The regimen used for *H. pylori* eradication is used for NHPH eradication. Most cases of NHPH infection in Japanese people are treated with triple-agent bacterial eradication therapy including a proton pump inhibitor, amoxicillin, and clarithromycin. The treatment lasts for 7–14 days [17–20]. In the present study, the patient received 7-day bacterial eradication treatment using three oral agents (vonoprazan fumarate, amoxicillin hydrate, clarithromycin) for *H. pylori* infection that are covered under the Japanese health insurance system. Both *H. pylori and H. suis* were successfully eradicated.

In Japan, the decrease in the number of patients with *H. pylori* infection is associated with a relative increase in the incidence of *H. pylori*-negative gastric MALT lymphoma. Consequently, the importance of diagnosing NHPH infection is expected to increase.

In summary, to the best of our knowledge, this is the first case of *H. pylori*-positive gastric MALT lymphoma with MLP-type lesions and *H. suis* superinfection in the background mucosa, which may have influenced the specific morphology of this case.
